# The milk fat globule size governs a physiological switch for biofilm formation by *Bacillus subtilis*

**DOI:** 10.3389/fnut.2022.844587

**Published:** 2022-08-11

**Authors:** Chen Raz, Margarita Maggie Paramonov, Moshe Shemesh, Nurit Argov-Argaman

**Affiliations:** ^1^Department of Animal Sciences, The Robert H. Smith Faculty of Agriculture, Food and Environment, The Hebrew University of Jerusalem, Jerusalem, Israel; ^2^Department of Food Sciences, Institute of Postharvest Technology and Food Sciences, The Volcani Institute, Agricultural Research Organization, Rishon LeZion, Israel

**Keywords:** Milk fat globule, *Bacillus subtilis*, biofilm formation, probiotic *Bacilli*, polar lipid, phosphatidylethanolamine

## Abstract

Milk lipids are organized in the form of milk fat globules (MFG), ranging in size from 0. 1 to 15 μm. The MFG size is closely associated with the composition of fatty acids, polar lipids, sphingolipids, cholesterol and the content of the MFG membrane (MFGM). Also, the MFGM integral proteins and glycoconjugates differ in composition and structure between different MFG size groups. These compositional differences may modulate the functionality of the MFG and its interaction with microbial cells. We report that small (2.3 μm) MFG facilitates the growth of the Gram-positive bacterium *Bacillus subtilis* whereas induction of biofilm formation was found in the presence of large (7.0 μm) MFG. Attempting to distinguish between the role played by the size from that played by the composition of the MFG, we compared phospholipid composition between treatments. We found that adjusting the phosphatidylethanolamine (PE) level to the concentration found in the small MFG, increased growth but suppressed biofilm formation in the presence of large MFG. The same normalization protocol for phosphatidylinositol (PI) or sphingomyeline (SM) did not exert a similar effect, suggesting a specific role for PE in regulating bacteria proliferation. We suggest that the content of MFGM, affected by MFG size, governs the ability of *B. subtilis* to utilize lipids from milk fat. This process might affect the bacterial decision-making toward biofilm formation or growth.

## Highlights

- The Small MFG Triggers the *B. Subtilis* Growth.
- The Large MFG Induces Biofilm Formation.
- Phosphatidylethanolamine Activates Growth and Suppresses Biofilm Formation of *B. Subtilis*.

## Introduction

As a complex nutritional and colloidal fluid, milk provides nutrients to a variety of different organisms, including microorganisms. Many microbial species produce an array of enzymes to digest milk constituents, and utilize the digestion products for cellular metabolism and growth (1–3). Hence, microbial growth in milk is usually studied in terms of quality, functionality, and safety of dairy products (4, 5). It was previously shown that thermal treatments like pasteurization reduce the bacterial load in dairy products and especially that of *Bacillus subtilis* (6). Dairy-associated bacterial species develop intricate interrelationships with milk components, characterized by either positive or negative outcomes in terms of food quality and functionality (7).

Studies on the interactions between bacteria and milk lipids have focused mainly on the antimicrobial effects of specific lipid molecules. For instance, Sprong et al. (8) reported a bactericidal effect of medium-chain fatty acids derived during triglyceride (TAG) hydrolysis. Beck et al. (9) showed that *Staphylococcus epidermidis* biofilm formation is mitigated by the sphingomyelin (SM) digestion product, sphingosine. When used as a supplement to bacteria substrate, short- and medium-chain fatty acids have been shown to affect physiological traits of Gram-positive *Bacilli* (3, 10). More specifically, butyric acid was shown to be a potent trigger for biofilm formation in *Bacillus* species, including *B. subtilis* (3).

Among the most common bacteria found in dairy farms and processing plants are the *Bacillus* species (11, 12). *Bacillus* species are the predominant Gram-positive bacteria isolated from both raw and pasteurized milk (11, 13, 14). Recently, the use of *Bacillus* species and in particular, *B. subtilis*, as probiotics has garnered much interest (15). *Bacillus* species have been reported to reduce the likelihood of developing respiratory infections and gastrointestinal disorders and as an adjunct to antibiotic use, to help overcome symptoms associated with irritable bowel syndrome (15–17). Therefore, the food industry, and especially the dairy sector, is pursuing means to deliver viable probiotic *Bacilli* to consumers. One of the significant challenges is improving the survivability of this bacterium during industrial processing and storage of milk and dairy products. Hence, understanding of the relationship between the bacteria and milk components is warranted.

The study of the interaction between bacteria and lipid molecules in milk has been mostly done using specific isolated lipid molecules from milk (3, 9, 10). However, milk lipids are rarely found in their free form (18). Instead, milk lipids are secreted in a complex macrostructure, termed milk fat globule (MFG), consisting of a TAG core covered with three layers of phospholipids (PL; (19)). The outer bilayer, termed MFG membrane (MFGM; (20)), originates from the plasma membrane of the milk producing cells, which envelopes the MFG during its secretion. The MFGM consists of 20–60% glycoproteins and 33% glycerophospholipids, primarily phosphatidylcholine (PC) and SM, with more minor contents of phosphatidylethanolamine (PE), phosphatidylinositol (PI), and phosphatidylserine (PS) (21). It has been suggested that the MFGM may elicit antagonistic activity against pathogens; in this regard, supplementation of MFGM in adults alleviated infection upon oral challenge with a diarrheagenic *Escherichia coli* (22), and reduced gut colonization of *Listeria* species in adult rats (23).

What is the specific most bioactive component of the MFGM is difficult to determine. Numerous studies have shown that carbohydrate moietiesof membranes glycoconjugates serve as a docking site for bacteria by recognition elements, like lectins. This interaction is also implied for the MFGM (24, 25) as significant amount bacteria adhere or preferably found in the vicinity of MFG when incubated with milk (reviewed by Douëllou et al. (26)). Although compelling evidences were provided to the interaction between MFG and bacteria, the role played by MFG size in this interaction has been rarely studied. As the MFGM content is determined by the MFG size, MFG size may determine the bioactivity of MFG and possibly its interaction capacity with bacteria.

MFG are secreted in a wide range of sizes, from 200 nm−15 μm (27, 28). MFG composition and size are tightly associated. For example, compared to large MFG, small MFG have a higher ratio of polar lipids to TAG because more membrane material is required to envelop the small MFG (29). This structural difference determines the composition of fatty acids (30), polar lipids (31) and proteins (32), and can modulate the MFG functionality and its interactions with bacteria.

Given that size governs the physical and chemical properties of intact MFG, we hypothesized that variations in MFG size would affect the physiological traits of bacterial cells. Accordingly, we characterized the response of the model bacterium *B. subtilis* to MFG size variations and compared it to the response to Ultra high temperature (UHT) milk or skim milk, as a control. We specifically focused on the lipid composition of the different sizes of MFG. Our results show that interaction of *B. subtilis* with the diverse-sized MFG fractions results in differential effects on bacterial growth and biofilm formation. Moreover, we discovered that polar lipids can regulate the transition of bacteria to a biofilm mode of growth.

## Materials and methods

### Bacterial strains and growth conditions

Wild-type cells of *B. subtilis* NCIB 3610 were grown in LB broth (10 g of tryptone, 5 g of yeast extract, and 5 g of NaCl per liter) or LB broth solidified with 1.5% agar (Difco, Le Pont de claix, France). For the negative control, *B. subtilis* was maintained in PBS (Sigma, Rehovot, Israel), skim milk 1.5% (1.5 g/ml powder in double-distilled water [DDW]; Difco), or UHT milk (local grocery store, 3% fat, 4.9% lactose, 3.3% protein, w/v). For colony-type biofilm formation, *B. subtilis* was grown on agar plates containing the biofilm-promoting medium LBGM (33). For bundle type biofilm formation, we used *B. subtilis* YC189 strain which harbors gene coding to cyan fluorescence protein (CFP) under the control of tapA promoter, which is the operon responsible for extracellular matrix synthesis of *B subtilis*.

### Cells visualization and counts

Bacteria were incubated with large or small MFG, or skim milk as control, for 5 h at 37°C at 50 RPM. After incubation, substrate was centrifuged at 10,000 RPM for 2 min, supernatant was discarded, pellet was resuspended, and 25 μl were loaded on a glass slip for visualization under fluorescence microscope in order to obseve the bacterial bundle biofilm formation. For each experiment, the initial number of cells was determined by colony forming units (CFU) after incubation of cells for 5 h in 37 °C at 50 RPM. In all experiments, CFU was calculated by inoculating 9 cm Petri plates with Luria broth (LB) agar with 100 μl of bacteria medium.

### MFG separation and normalization of fat content

MFG were separated using the protocol of Ma and Barbano (34). Raw milk samples were collected from the dairy farm of the Agricultural Research Organization's Volcani Center (Israel) and brought to the laboratory on ice. The milk was run through a 70-ml separative column overnight at 16°C. The bottom fraction (F1) and top fraction (F7) were collected into different tubes, and taken for further analysis. The amount of milk solids (fat, protein, and lactose) in the small and large MFG fractions was measured by infrared methodology (Lactoscan; Novazagora, Bulgaria). At the beginning of each experiment, the fat percentage was normalized by diluting the large MFG fraction with PBS to obtain fat concentrations similar to those in the small MFG fraction (Table 1).

**Table 1:** Percentage of milk solids and content of phospholipids in the small and large MFG fractions before normalization and after normalization to fat content (dilution of the large MFG fraction).

	**Small MFG fraction**	**Large MFG fraction**	**Large MFG after dilution**
Fat (%)	1.4 ± 0.5	26.4 ± 7	1.38 ± 0.3
Protein (%)	3.9 ± 1.6	8.9 ± 2.6	0.46 ± 0.13
Lactose (%)	4.6 ± 1.3	7.3 ± 1.8	0.4 ± 0.1
Phospholipids (μg/ml)	407 ± 186	2,149 ± 142	113.1 ± 14
Surface area of a MFG (μm^2^)^2^	21.8 ± 7.2	159.5 ± 41.3	
CFU per surface unit ^3^	7,523 ± 1551	2,232 ± 464	

^1^ MFG, milk fat globule.

^2^ Surface area was calculated using the formulation: 4πr^2^.

^3^ This value was calculated as the CFU after 24 h of incubation with small or large MFG divided by the total surface area of each treatment.

### Experimental design

To characterize the interactions between bacteria and MFG*, B. subtilis* cells were incubated for 24 h at 23°C in the presence of either small or large MFG substrate that was normalized for fat content (small or large MFG treatment, respectively). Controls were skim milk and UHT milk which represent similar contents of protein and lactose, respectively. Time points were selected to assess the effect of MFG size on bacterial growth. For experiments examining colony-type biofilm formation, *B. subtilis* cells were grown at 37°C with shaking at 50 rpm for 5 h on agar plates supplemented with a mixture of LBGM and the milk sample. All experiments were conducted in duplicates, and each experiment was performed at least twice independently.

### MFG staining

A 1-ml aliquot of milk collected from each fraction (F1 and F7) was stained with Nile red (dissolved in acetone, 42 μg/ml; Sigma, Rehovot, Israel) for 2 h at room temperature. For fixation, agarose was dissolved in DDW (5 mg/ml) and mixed with the milk sample and the staining dye in a 1:20 (v/v) ratio. The samples were visualized under an Olympus BX40 fluorescence microscope equipped with an Olympus DP73 digital camera using Callens Entry software (version 1.7; Olympus, Tokyo, Japan). Lipid droplet diameter was measured using ImageJ software (version 1.48, NIH, Bethesda, MD). Milk fat was characterized by small (2.3 ± 0.03 μm) and large (7 ± 0.1 μm) MFG. For each MFGsurface area was calculated using the formulation: 4Πr^2^. The total surface area of each treatment was calculated as the surface area multiplied by number of lipid droplets after normalized for fat content. This value was used to calculate the number of CFU per surface unit for small or large MFG.

The ratio between total surface area of small compared with large MFG treatment was calculated according to the following formula:

Ratio of surface area= (4π r_small_^2*^ #small MFG) / (4π r_large_^2*^ #large MFG)
r_small_ is the average radius of small MFG.
r_large_ is the average radium of large MFG.
#small MFG is the number of MFG in a sample of small MFG treatment.
#large MFG is the number of MFG in a sample of the large MFG treatment after normalizing for total milk fat content.

### Lipid extraction and chromatographic analysis

For the lipid analysis, we used analytical reagent-grade petroleum ether (Gadot Lab Supplies, Netanya, Israel), sulfuric acid (H_2_SO_4_; Diagnostic Products, Los Angeles, CA), chloroform, methanol, and ethanol (Purity 99%, Bio-Lab, Jerusalem, Israel), and dichloromethane and methanol for liquid chromatography (Purity 99.8%, Merk KGaA, Darmstadt, Germany). Effects of the individual membrane components PE, SM and PI (Sigma, Rehovot, Israel) or petroleum ether (Gadot Lab Supplies, Natanya, Israel) were tested on bacterial proliferation.

Suspensions of inoculated bacterial cells and MFG fractions (including lipids from the bacteria and specific milk fractions) were extracted by the Folch method (35). Briefly, each sample (t = 0 h, t =24 h) was incubated for 1 h with Folch mixture (chloroform:methanol, 1:2 v/v). The organic phase was separated by the addition of DDW and overnight incubation at 4°C. The upper phase was then removed and the lower phase was filtered through glass wool. The lower phase was evaporated under nitrogen and then dissolved in chloroform:methanol (3:97, v/v). Samples were kept at −20°C until further analysis. Identification and quantification of polar and neutral lipids were performed by HPLC (HP 1200, Agilent Technologies Santa Clara, California) analysis combined with an evaporative light-scattering detector (ELSD; Agilent) as previously described (30). The separation process was managed by ChemStation software (Agilent) which permitted data acquisition from the ELSD. Lipid identification was enabled by the use of external standards; triacylglycerol, diacylglycerol, monoacylglycerol, free fatty acids, cholesterol, PE, PI, PS, PC, and SM. Standard curves were used to calculate weight percentage of each of the identified phospholipids and cholesterol from the overall weight of the identified membrane components (i.e., cholesterol, PE, PS, PI, PC, SM), as indicated below.

### HPLC/ELSD calibration

The identification of phospholipids and SM was carried out by comparison with the retention time of pure standards. To evaluate phospholipids and SM, five calibration curves were determined from the area values obtain by injecting different amounts of standards: cholesterol (2–15 μg), free fatty acids (5–80 μg), PE (2–25 μg), PI (5–80 μg), PC (0.5–10 μg), and SM (5–80 μg). Calibration curves were calculated by applying the equations of the power model to the area and concentration values; cholesterol: y = 0.1016X^0.49^ (r^2^ = 0.97), free fatty acids: y = 1.73X ^0.41^ (r^2^ = 0.99), PE: y = 0.035X^0.63^ (r^2^ = 0.99), PI: y = 0.114X^0.607^ (r^2^ = 0.99), PC: y = 0.051X^0.603^ (r^2^ = 0.99), SM: y = 0.11X^0.62^ (r^2^ = 0.99). The sum of glycerolphospholipid, SM and cholesterol concentrations was regarded as total MFGM polar lipids weight (100%). Each of the polar lipids' weights in 1 mL of milk was calculated according to the standard curves, and its weight percentage from the sum of detected and identified lipids was determined (Supplementary Table S1).

### Testing the effect on bacterial growth and biofilm formation of specific constituents of the MFGM

To examine the effect of MFGM PL composition on *B. subtilis*, we added specific phospholipids (PE, PI and SM) to the large MFG treatment to match their concentration in the small MFG treatment. First PE concentration was adjusted to a concentration of 25% of total polar lipids, to match its concentration in small MFG (31). After incubation bacterial growth was assessed according to CFU. Biofilm formation was examined using CFP-tagged *B. subtilis* or seeding the bacteria on the agar substrate of MFG mixed with different PLs. To evaluate if the effect of PE supplementation was specific to PE or can be achieved with other PL, we supplemented the large MFG treatment with same ratios of PI and SM.

### Analysis of bacterial survival after incubation with MFG

Samples containing *B. subtilis* suspensions generated as described above were grown for 0, 2,4,6, 8, 12 and 24 h aerobically at 37°C with shaking at 150 rpm. The samples were then subjected to CFU counting on LB agar plates.

### Survival rates during heat treatment

To evaluate the number of colonies derived from spores, the *B. subtilis* cells were incubated with the MFG fractions for 24 h at 23°C with shaking at 150 rpm. Then, heat killing was performed at 80°C for 20 min. Cell numbers after heat killing were quantified by the CFU method using LB agar plates.

### Visualizing *B. subtilis* interactions with MFG by confocal laser scanning microscopy

*B. subtilis* expressing green fluorescent protein (GFP;YC161) was incubated with the MFG fraction for either 5 h at 37°C with shaking at 50 rpm, or for 24 h at 23°C with shaking at 150 rpm. The MFG were stained with Nile red as described above. A 25-μl aliquot of each sample was placed on a glass microscope slide and visualized in a transmitted light microscope using Nomarski differential interference contrast (DIC) and 488 nm laser for CFP excitation (Leica, Wentzler, Germany). To visualize biofilm formation, *B. subtilis* cells expressing CFP (YC189) were incubated with the MFG fraction for 5 h at 37°C with agitation at 50 rpm. A 25-μl aliquot of each sample was placed on a microscope slide and visualized in a transmitted light microscope with Nomarski DIC with 458 nm laser for CFP excitation.

### Statistical analysis

All statistical procedures were performed using JMP software version 14.0 (SAS Institute, Cary, NC). Data are means ± SE. Comparisons between treatments were submitted to ANOVA followed by LS Mean Tukey–Kramer HSD multiple-comparison test. Differences among time points on the bacterial growth curve were tested by Tukey–Kramer multiple comparison test. Values are presented as mean ± SE. Significance level was set at 0.05.

## Results

### Lipid composition of MFG depends on their size

**Figure 1:**
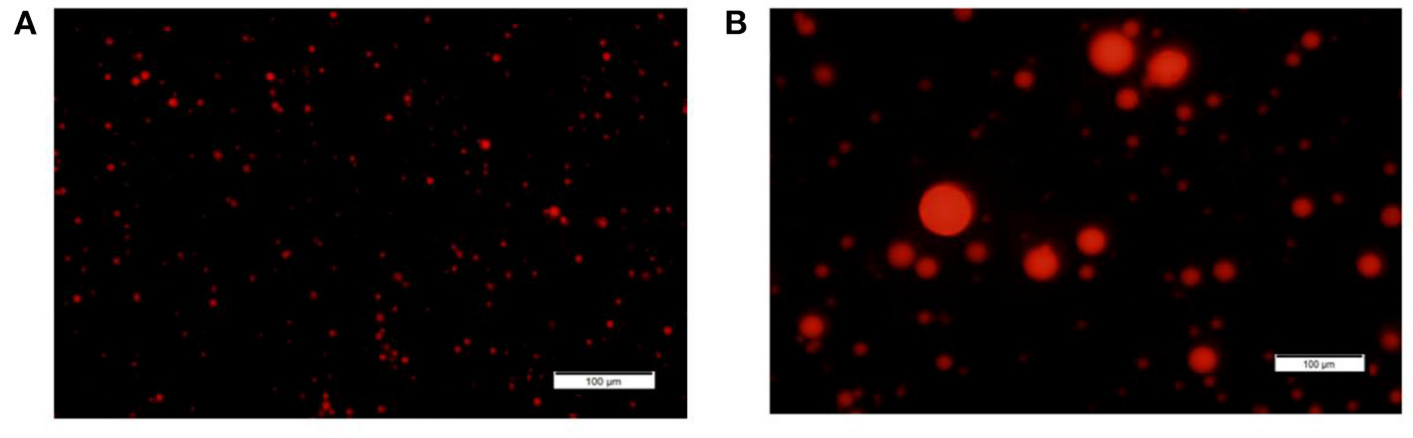
MFG size fractions. Representative images of small and large MFG fractions stained with Nile red and visualized under fluorescence microscope [magnitude X20, **(A, B)**, respectively].

Based on our initial hypothesis of a possible link between MFG composition and size, we chose to characterize two MFG size groups: large (7 ± 0.1 μm) and small (2.3 ± 0.3 μm; Figures 1A,B, the respectively). A relative surface area of small MFG was 7.88 fold higher compared with that of large MFG. While the total fat was higher by approximately 20 fold between treatments, the phospholipid content was almost similar, attributed to the higher surface area in small compared with large MFG (Table 1).

### Interaction of bacteria with MFG affects their growth

**Figure 2:**
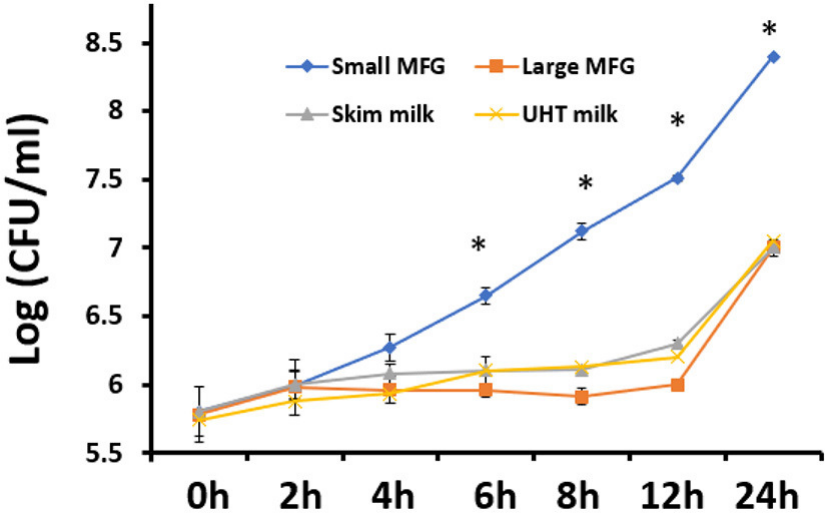
The effect of MFG size on growth of *B. subtilis*. CFU quantification of *B. subtilis* growth during incubation with small or large MFG during 24 h of incubation, compared with skim milk and UHT milk as controls. asteriks reresent significant differences between treatments at specific sampling time (*P* ≤ 0.05).

It was further hypothesized that the differences in MFG composition would affect the bacteria's growth rates. Thus, we investigated the possible effect of MFG size, on bacterial growth during incubation of *B. subtilis* cells in the presence of either small or large MFG. After 24 h of incubation (Figure 2), we observed 100-fold increase in the number of *B. subtilis* cells incubated with small MFG whereas in the large MFG, skim and UHT treatments only a 10 fold increase was observed after 24 h (*P* ≤ 0.05). Differences in cell number between the small MFG treatment and all other treatments were observed starting from 6 h of incubation. To understand what is the impact of the surface area available for the interaction of bacteria and MFG, we calculated the CFU per surface area unit (Table 1). The results show that per surface area unit, the bacteria growth was much enhanced by the small MFG. Hence, the differences in size of the large and small MFG play a role in regulating growth or biofilm formation of the bacteria.

### Modulation of lipid profile following *B. subtilis* interaction with MFG of different sizes

**Figure 3:**
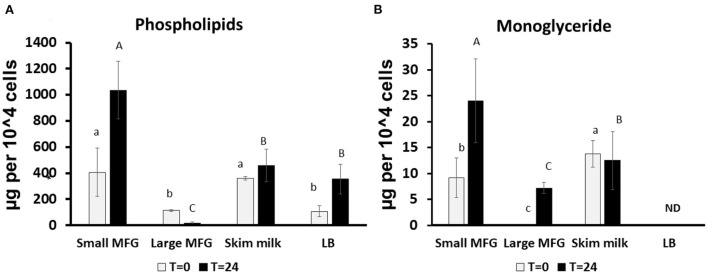
Changes in lipid profile following incubation of *B. subtilis* cells with small or large MFG. **(A)** Quantification of the sum of phospholipids after incubation of *B. subtilis* with different MFG sizes compared to skim milk or LB broth bacteria substrate. **(B)** Quantification of MG following incubation of *B. subtilis* with MFG size fractions compared to skim milk or LB substrate. Lowercase letters indicate significant differences between treatments at t = 0 (white bars; *P* < 0.05). Uppercase letters indicate significant differences between treatments at t = 24 h (black bars; *P* < 0.05). ND indicated under detectable levels.

We further performed lipid profiling following incubation of *B. subtilis* with small or large MFG. The analysis was performed at 0 and 24 h of incubation on the mixture of MFG and bacteria because it was impossible to segregate the bacteria from the substrate due to their close interaction after 24 h of incubation (Supplementary Figures S1, S2 for small and large MFG, respectively). Compared with the lipid composition at the beginning of incubation, after 24 h, the small MFG increased the polar lipid amount by 2.5-fold (*P* < 0.05; Figure 3A), which may be attributed to enhanced bacteria proliferation. On the other hand, after incubation with large MFG, PL content decreased significantly. This result can be attributed to the digestion of polar lipids from the MFG by the bacteria without significant increase in theier numbers. This assumption is further supported by the increase in the concentration of monoglycerides (MG) (Figure 3B), in both small and large MFG, which may originate partially from milk degradation. It should be noted that in the LB treatment no MG were detected at the beginning or the end of the incubation, which is in accordance with the fact that LB does not contain any significant amount of fat.

### Interaction of *B. subtilis* cells with MFG affects bacterial biofilm formation

**Figure 4:**
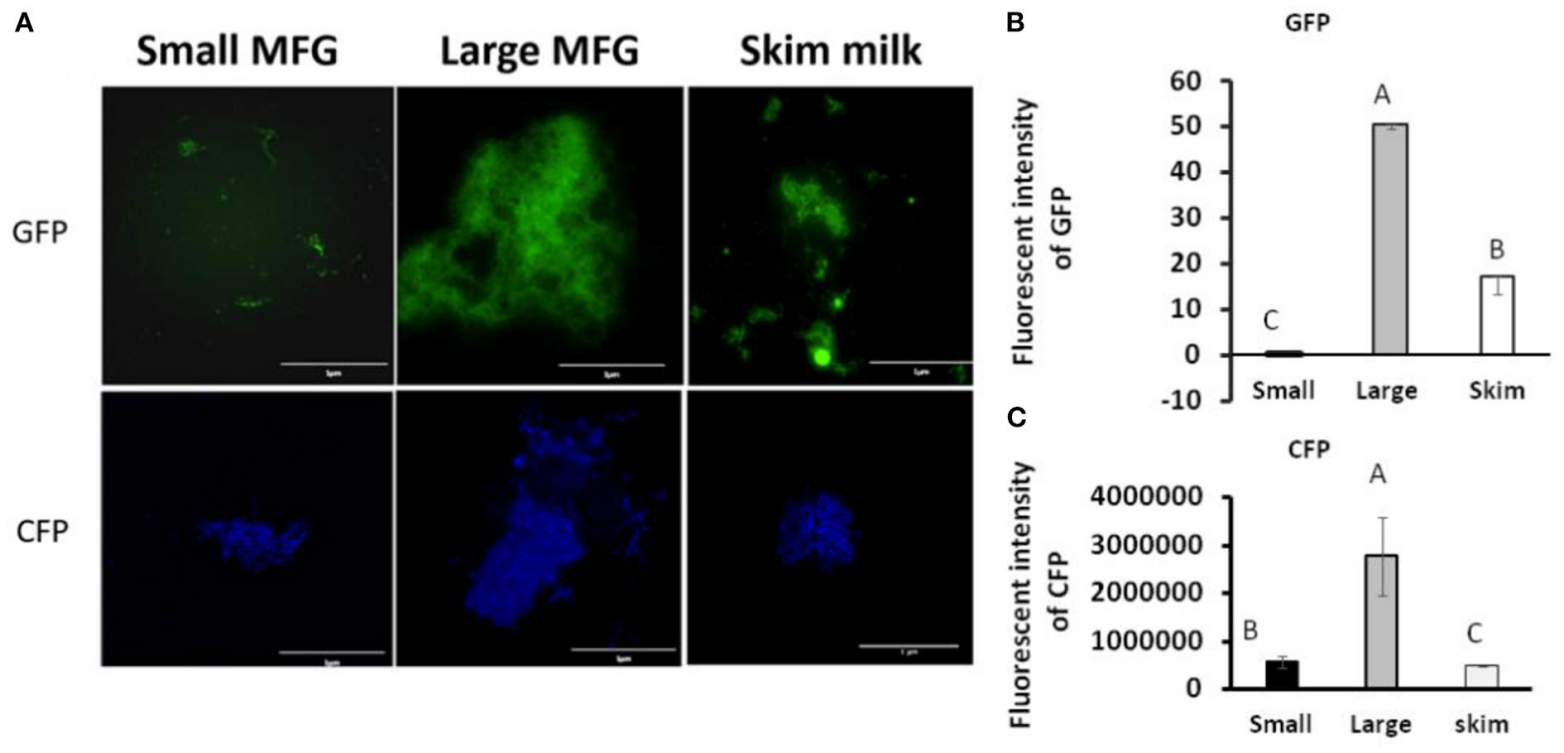
MFG size affects biofilm formation by *B. subtilis*. **(A)** representative image of *B. subtilis* tagged with GFP (upper row, green) and CFP (bottom row, blue) reporter incubated with either small or large MFG compared to incubation in skim milk as a control. **(B)** Integrated fluorescence intensity of GFP and **(C)** CFP. The fluorescence density was measured using ImageJ. Values represent means of 2 independent experiments, with 3 replicates for each treatment (*n* = 3). Different letters represent significant differences between treatments (*P* ≤ 0.05).

We further examined the consequences of the MFG–bacterium interactions on biofilm formation. Using GFPtagged cells, increased bacterial bundling was observed in the presence of large MFG (Figure 4A, upper panel); in addition, we found notable upregulation of CFP-tagged operon *tapA* expression (Figure 4A, lower panel): a 25-fold increase in the fluorescence intensity when bacteria were incubated with large compared to small MFG (*P* < 0.05; Figures 4B,C), whereas intermediate values were obtained when bacteria were incubated with skim milk as a control.

**Figure 5:**
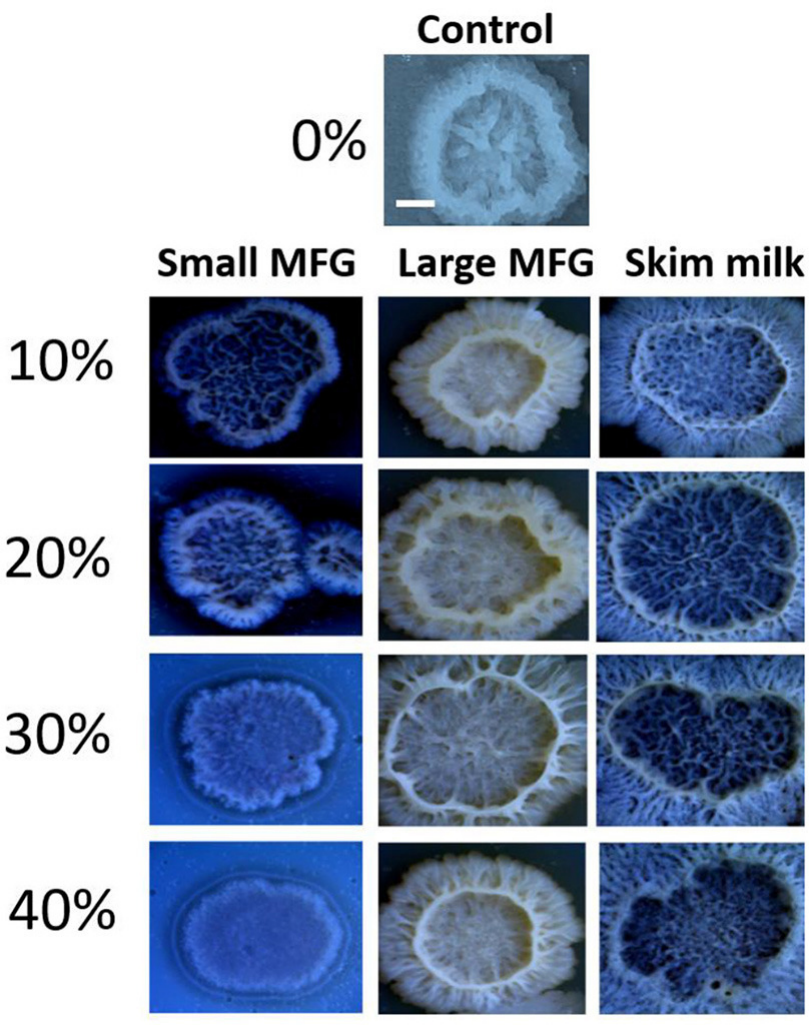
Size of MFG incorporated into a solid substrate affects the structure of biofilm colonies formed by *B. subtilis*. LBGM plate with no milk fraction was used as a control. Scale bar represent 3 mm. Different concentrations (v/v) of small or large MFG were incorporated into agar to form a solid substrate to study colony-type biofilm formation.

Next, the effect of MFG different size on colony-type biofilm formation was determined. We used LBGM agar plates substrate supplemented with different doses of either small or large MFG (10–40% mixed in agar, v/v). We found a dose-dependent response to the different concentrations of small MFG in the substrate. Whereas inclusion of large MFG in the substrate enabled the formation of large, well-developed colonies, with a complex network of channels, inclusion of small MFG in the substrate resulted in much less developed colonies, with much lower surface area, much less developed 3D structure and a much more homogeneous structure (Figure 5). The large MFG treatment was not significantly different from skim milk, indicating that the significant effect stems from inhibition by the small MFG rather than enhancement by the large MFG (differences in color of the image are a result of automatic adjustment of intensity of the imaging software).

### The role of polar lipids in modulating growth and biofilm formation of *B. subtilis*

**Figure 6:**
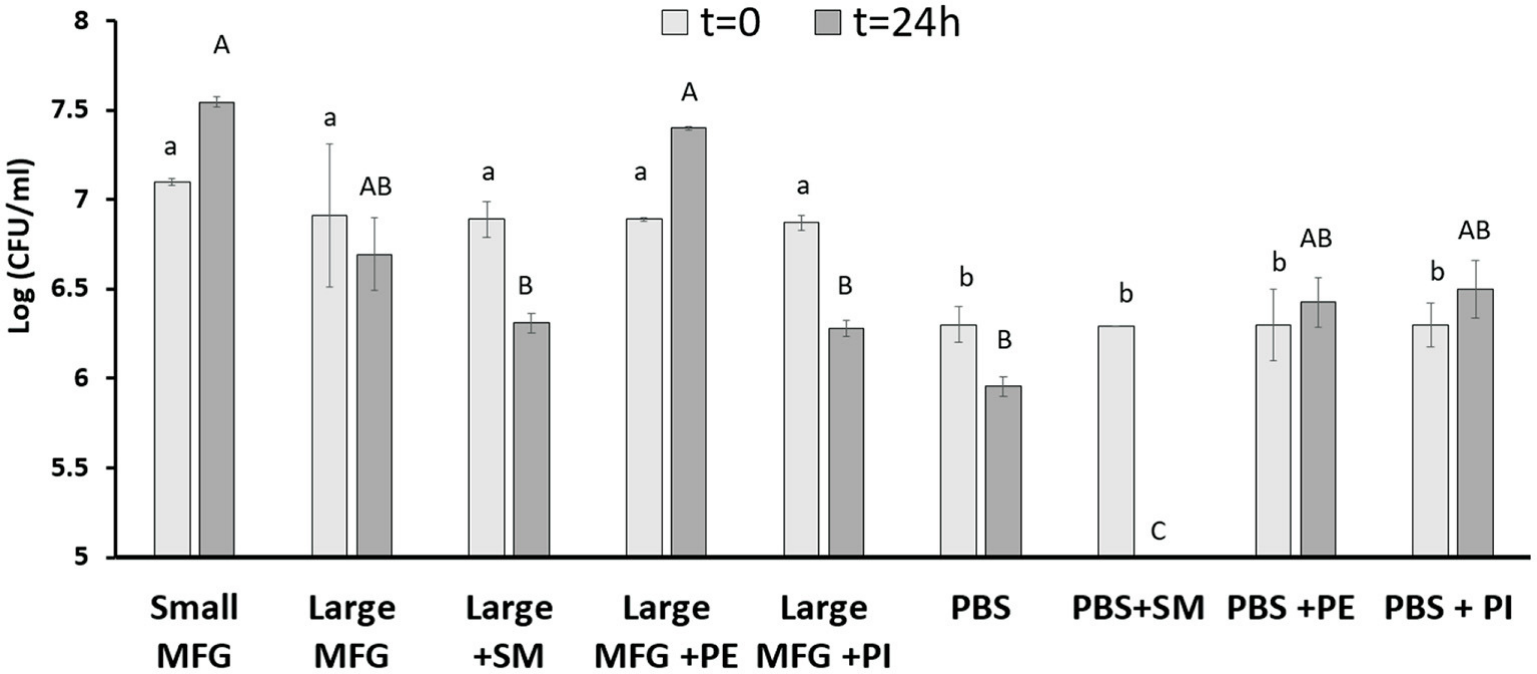
Effect of MFG size and supplementation of polar lipids on *B. subtilis* growth. CFU counting of *B. subtilis* after 0 and 24 h of incubation with small or large MFG, or large MFG supplemented with PE, PI or SM to match their concentrations in the small MFG fraction. Uppercase letters indicate significant differences between the treatment groups at 24 h whereas lowercase letters indicate significant differences between the treatment groups at 0 h (*P* ≤ 0.05).

It was further hypothesized that differences in the MFGM polar lipid composition play a role during the interaction of bacterial cells with either small or large MFG. We then added PE and SM to the large MFG treatment to match their concentrations in the small MFG treatment. In addition, to study if the effect is ubiquitous for all PL, we added PI and SM to the large MFG sample (both added to achieve similar concentrations in MFGM of small and large MFG). PBS and PBS supplemented with SM, PI or PE was used as a control (Figure 6). The inclusion of MFG (large or small) in the media, enhanced bacteria growth at time 0, as indicated by greater counts of every medium containing milk fat, compared with the media containing PBS. However, 24 h after incubation with the different media, marked differences were noted according to the MFG size used and their supplementation. For example, supplementation of PE to the large MFG sample resulted in a similar proliferative effect as that exerted by the small MFG (Figure 6). This proliferative effect was not seen when SM or PI were supplemented to the large MFG treatment. Interestingly, the effect of PBS+PE did not differ from that of PBS alone or of PBS supplemented with PI, meaning that the pro-proliferative effect of PE is only manifested in the presence of other milk lipids (Figure 6). On the other hand, the supplementation of SM to PBS completely eliminated bacterial growth, exerting a significant bactericidal effect, which is somewhat suppressed when the SM is embedded in the MFGM.

**Figure 7:**
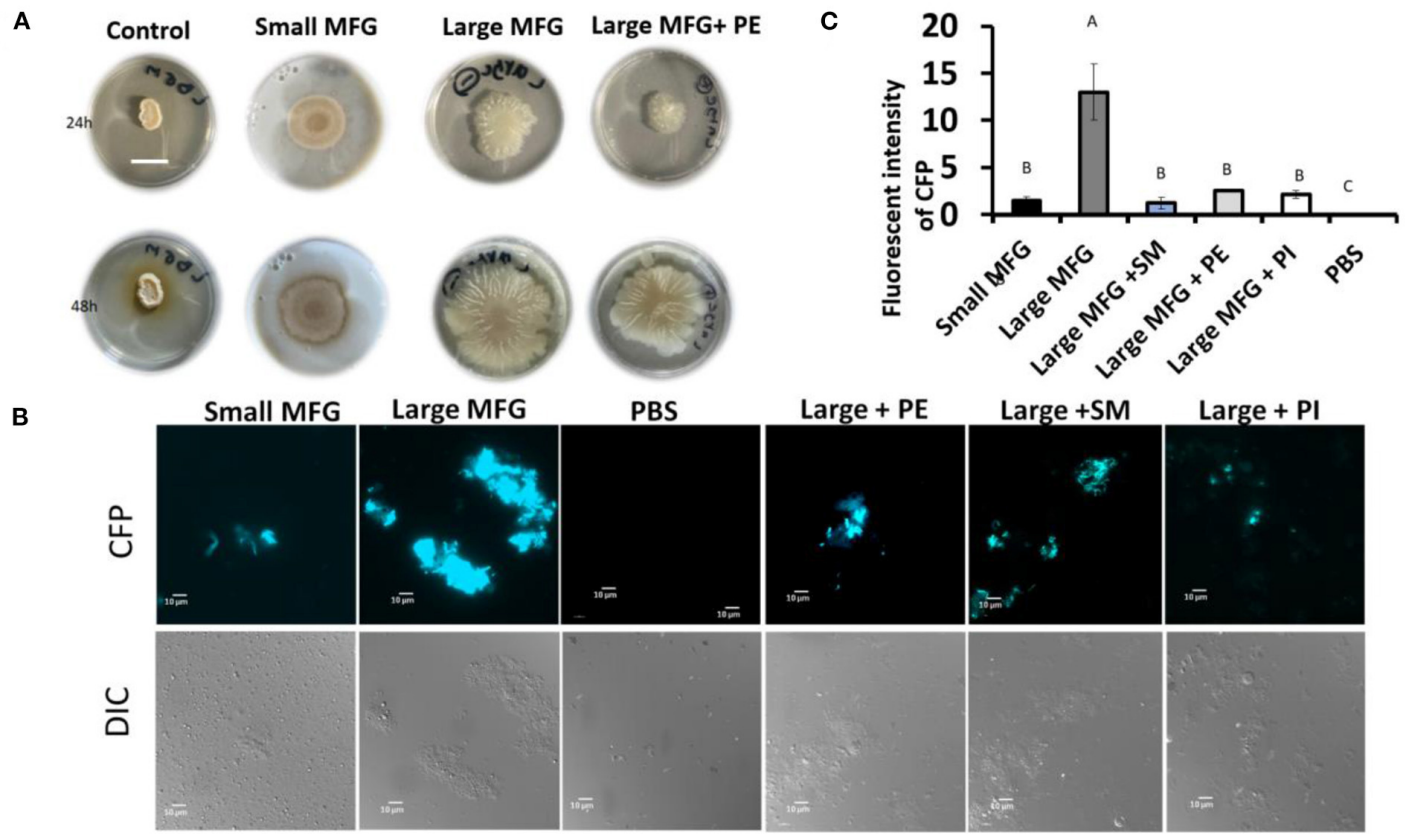
Effect of supplementation of polar lipids on biofilm formation. Colony- or bundle-type [**(A, B)** respectively] biofilms of *B. subtilis* grown on LBGM mixed with PBS or MFG of different sizes, compared to large MFG supplemented with PE, SM or PI in amounts matching those in the small MFG fraction. Addition of the MFGM PL (PE, SM, PI) to the large MFG (for comparison with the small MFG) a showed inhibited biofilm formation: **(A)** representative image of bacteria growth on LBGM mixed with milk fractions as substrate (1:1) Scale bar represent 1.5 mm. The results indicate inhibitng of the colony type biofilm formation after incubation with the small MFG fractions and with large MFG with addition of PE. **(B)** representative image of cells was grown for 5 h, 37°c and 50 RPM, and analyzed by fluorescent microscopy **(C)**, relative fluorescence of the bundle-type biofilm was quantified. values are mean±standard errors of 2 independent experiments with 3 replicates for each treatment in each experiment. Uppercase letters indicate significant differences between the treatment groups (*P* ≤ 0.05).

Furthermore, while testing the effect of supplemented polar lipids on biofilm formation, we found that the biofilm-inducing effect of the large MFG was abolished upon addition of PE, resulting in a phenotype similar to that obtained when the bacteria were incubated with small MFG (Figure 7A). However, unlike PE's growth-inducing effect, the biofilm-inhibiting effect was not specific for PE; biofilm formation was abolished with every polar lipid addition to the large MFG treatment, i.e., PI, SM, and PE (Figures 7B,C).

## Discussion

The effect of milk lipids on bacterial metabolism, growth, and biofilm formation has been extensively studied using free fatty acids and specific polar lipids (3). However, the natural secretion process of milk lipids is in the form of MFG, with a low abundance of free fatty acids. The role of this structure, and how bacterial metabolism and growth are affected by its complexity, remain to be determined. Here we used MFG in their native, complex form to study their effect on physiological processes occurring in bacteria. We found that bacterial growth and biofilm formation is affected by the MFG size. This modulation can be driven by the differences in lipid components and relative abundance between large and small MFG, and also by other bioactive components associated with the MFGM, like glycoproteins.

MFG size is closely associated with the composition of polar lipids, fatty acids (29, 36), and proteins (32). The mass ratio between the MFG components, specifically the TAG core, and the MFGM differ in MFG of different sizes. The MFGM is a source of membrane polar lipids and glycoconjugates like glycolipids and glycoproteins, that are used by different bacteria strain as adhesion sites (26). These adhesion molecules could affect the response of *B. subtillis* to the large compared with small MFG recorded in the present study. However, comparing bacteria number (CFU) after incubation with MFG, indicated that for the same surface area, the bacteria growth was much enhanced by the small MFG. These results suggest that the composition of the MFG plays a role in determining the growth rates of the bacteria, beyond simply providing more adhesion sites. For example, fatty acid composition differs between large and small MFG, and the large MFG typically is richer in short and medium chain fatty acids due to their greater TAG content. Some of these short-saturated fatty acids exert antimicrobial effect (3) and may also affect the production of biofilm by different bacteria strains (37).

Considering these differences between different sizes of MFG, we anticipated that the size of the MFG used as a substrate in the current study will affect the composition of fermentation products that would be released in the medium and consequently affect bacterial growth and biofilm formation. Accordingly, we found compromised growth when the bacteria were incubated with large compared to small MFG. This effect could be attributed to a higher concentration of short- to medium-chain fatty acids in the large MFG (23) that exert a bactericidal effect against Gram-negative and positive bacterial species (8, 38).

We also aimed to elucidate whether the concentration of lactose in the media is the factor enhancing bacteria growth in the small compared with large MFG. Therefore, we used UHT milk as a high lactose control compared to UHT milk, small MFG enhanced bacteria growth, suggesting lactose was not the main driver for enahced growth in the small MFG treatment.

In addition to a change in growth, butyrate has been shown to induce biofilm formation in *Bacillus* species (3). Butyrate is found mainly in the TAG core of the MFG and therefore is found in greater quantities in large compared to small MFG (39). The butyrate in the MFG is available to the bacteria since it was previously shown that *B. subtilis* can hydrolyze TAG and release short-chain fatty acids (36). Taken together, the lower content of TAG and hence butyrate in the small MFG may be reason for not detecting significant biofilm formation when the bacteria were incubated with small MFG (Figure 4). The presence of large MFG induced biofilm formation, as also evidenced by expression of the *tapA* operon-one of the major markers of biofilm formation (Supplementary Figure 1). Moreover, during the examination of colony-type biofilm formation, we observed increasing robustness of biofilm-associated structures, i.e., a heterogeneous structure including channels and towers, in the presence of large MFG; in contrast, exposure to small MFG seemed to inhibit biofilm formation in a dose-dependent manner (Figure 5). Consequent to the induced biofilm formation, large MFG inhibited bacterial growth (Figure 2). Accordingly, *B. subtilis* entering the biofilm-formation stage has been shown to exhibit periodic arrest of bacterial growth (40), to enable the utilization of nutrients for the existing cells which are already protected by the biofilm colony (41). This growth arrest may explain the lower concentration of phospholipids found after incubation of bacteria with large but not small MFG.

Another property of well-developed colonies is structural heterogeneity, which suggests different transcriptome of the bacterial subpopulations, especially of genes involved in metabolism associated with biofilm formation (40, 42). Accordingly, the formation of well-developed colonies when bacteria were exposed to large, but not small MFG implies differences in metabolic capacity and hence, differential capacity for milk lipid utilization. In accordance, we found differences in the lipid utilization by the bacteria when incubated with small or large MFG, as determined by comparison of the lipid profiles of the media, before and after incubation.

It should be noted that to exert their physiological effects on bacterial growth or biofilm formation, milk fatty acids first need to be released from their macrostructure, typically from their glycerol backbone. This process requires the hydrolysis of ester bonds, carried out by either endogenous lipases released into the milk by mammary gland cells (43, 44) or by lipases from an exogenous source. During storage, lipases from bacterial sources can break down ester bonds and release free fatty acids into the milk (45). In the present study, milk samples were pasteurized prior to their inoculation hence ensuring that the source of active lipases is the bacteria added to the samples (46).

The differential growth rates observed during the study can be explained by the fermentation kinetics of the different-sized MFG. Previous studies on digestion efficiency of lipid droplets, in the form of micelles, showed the digestion change according to the droplet size, as determined by measuring the accumulation of digestion products over time. It was found that emulsions with small lipid droplets (~0.7 μm) were digested more rapidly and efficiently than those with large lipid droplets (~10–15 μm; (47–50)). We hypothesize that the same digestion efficiency can be implied for the MFG in the present study and assume that small MFG are digested more efficiently. Digestion efficiency requires interaction between bacteria and surface area of the MFG. Given that small MFG has larger surface area compared with large ones (51), nutrient availability for bacterial growth could be higher in small compared with the large MFG which may explain the enhanced growth in the small MFG treatment. It should be noted that the treatments used in the present study were normalized to the fat content, but maintained differences in lactose and protein. To determine whether the induced growth of the small MFG treatment was not attributed to the lactose or protein treatment, a skim milk and UHT milk were used as controls. The results show that the induced growth was not a function of lactose or protein concentration but rather a change in the fat structure or size dependent composition.

In addition to specific fatty acids, the MFG have a polar lipid envelope, the MFGM. Although in most species the MFGM represents <10% of total milk lipids (52), its composition and content differ between large and small MFG (31, 39). The major constituents of the MFGM are polar lipids, especially PC, PE, PS, PI and SM (52). The concentration of PE is higher in small compared with large MFG (31). Moreover, small MFG has greater surface area compared with large MFG (51) and thus elements of the MFGM, such as PE and its digestion product, ethanolamine, are more available for the bacteria. In *B. subtilis*, PE is one of the major membrane constituents (53) which can explain the positive correlation found between PE concentration in the media and growth rate of bacteria (54). These findings suggest a central role for PE in membrane lipid synthesis and in *B. subtilis* division (55). Hence, its availability, or the availability of its precursor, ethanolamine, could contribute to bacterial metabolism, proliferation, and survival. These pro-proliferative effects of PE could explain the induced growth rate of *B. subtilis* in the presence of the small, but not large MFG. Therefore, the pro-proliferative effect of small MFG found in the present study could be supported by the capacity of *B. subtilis* to utilize PE more efficiently from them as compared to the large MFG.

To assess the role played by PE in directing bacteria toward proliferation vs. biofilm formation, we used a comparative approach; exogenous PE was added to the large MFG treatment to match the content of PE in the small MFG treatment. As a result, the phenotype of *B. subtilis* grown on a substrate of small MFG was mimicked by the large MFG fortified with PE, as manifested by enhanced growth and inhibition of biofilm formation (Figures 4, 5). To determine if the effect is specific to PE, we supplemented large MFG treatment with SM to match its concentration in the small MFG treatment (29, 31, 39), yielding different results: addition of SM to the large MFG treatment decreased bacterial proliferation compared with small MFG and large MFG treatments (without supplements). This result is in accordance with the finding that sphingolipids exert an antimicrobial effect on Gram-positive and negative bacteria (56). Supporting the unique effect of PE on proliferation are the results of the supplementation of PI to the large MFG treatment that did not alter proliferation compared to the large MFG alone. It should be noted that these phenotypes were all visualized only in the presence of milk fat. Taken together, our findings indicate that PE exerts a pro-proliferative effect in the presence of other MFG-related constituents.

The specificity of the polar lipids, in terms of their effect on the bacterial response to large or small MFG, was diminished when biofilm formation was assessed. Biofilm formation was inhibited upon addition of either PE, PI or SM to the large MFG treatment. While the biofilm-inhibiting effect of SM has been described previously (10), the authors are not aware of any prior documentation of biofilm inhibition by PE or PI.

To conclude, our results suggest that the small MFG provide greater availability of nutrients to the bacteria whereas the large MFG exert bactericidal effect. Thus, the size of the MFG as a substrate determines whether enhanced growth or biofilm formation will occur. In addition, we demonstrated a role for PE in the regulation of these processes, and that inhibition of biofilm formation can be achieved by supplementing different polar lipids.

## Data availability statement

The raw data supporting the conclusions of this article will be made available by the authors, without undue reservation.

## Author contributions

NA-A conceived. MS, NA-A, and CR designed the experiments and wrote the paper. CR and MP performed the experiments. CR and NA-A analyzed the data. All authors contributed to the article and approved the submitted version.

## Funding

This study was partially funded by the Israeli Ministry of Agriculture Chief Scientist, under contract number 12-04-0042. NAA is Baron de Hirsch Chair in Animal Husbandry.

## Conflict of interest

The authors declare that the research was conducted in the absence of any commercial or financial relationships that could be construed as a potential conflict of interest.

## Publisher's note

All claims expressed in this article are solely those of the authors and do not necessarily represent those of their affiliated organizations, or those of the publisher, the editors and the reviewers. Any product that may be evaluated in this article, or claim that may be made by its manufacturer, is not guaranteed or endorsed by the publisher.
