# Supplementary material for: The milk fat globule size governs a physiological switch for biofilm formation by Bacillus subtilis
Source: Front Nutr. 2022 Aug 11;9:844587. doi: 10.3389/fnut.2022.844587 (PMC9404525; doi:10.3389/fnut.2022.844587)
Supplement: Supplementary file 1 [file Data_Sheet_1.PDF]

Supplemented data file

Raz et al.

Table S1:

Weight % of the identified polar lipids in the small and large MFG treatments.

	PI	PE+PS	PC	SM
Small MFG	3.9±2.1	35.3±21.4	9.4±4.1	51.3±4.9
Large MFG	0.9±0.09	29.8±2.9	16.6±0.2	52.5±2.36

PI- phosphatidylinositol; PE+PS- the combined weight precent of phosphatidylethanolamine and phosphatidylserine; PC- phosphatidylcholine; SM- sphingomyelin.

Figure S1:

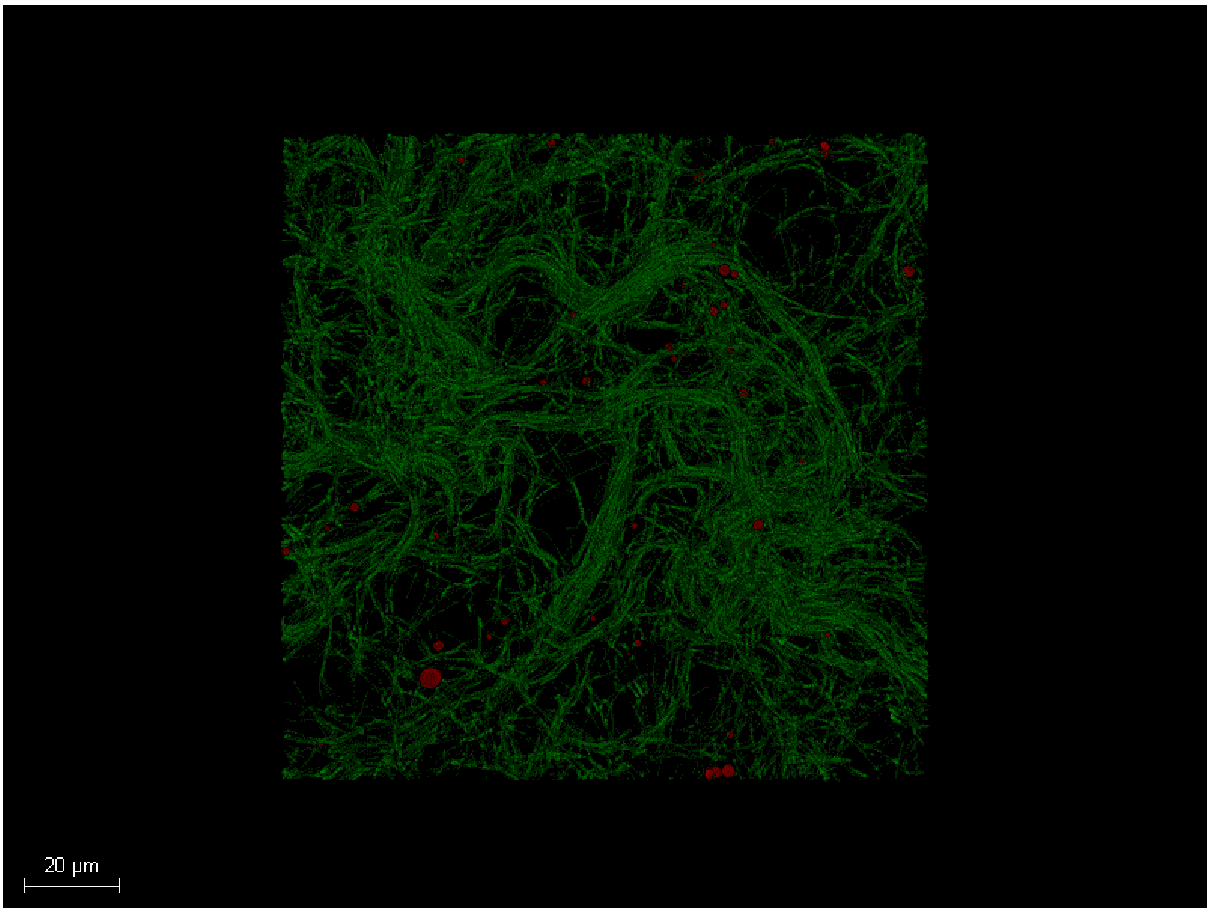

Confocal micrograph of *B. subtilis* expressing GFP (YC161) incubated with small MFG fraction for 24 h at 23ºC at 150 rpm. The MFG were stained with Nile red.

Figure S2:

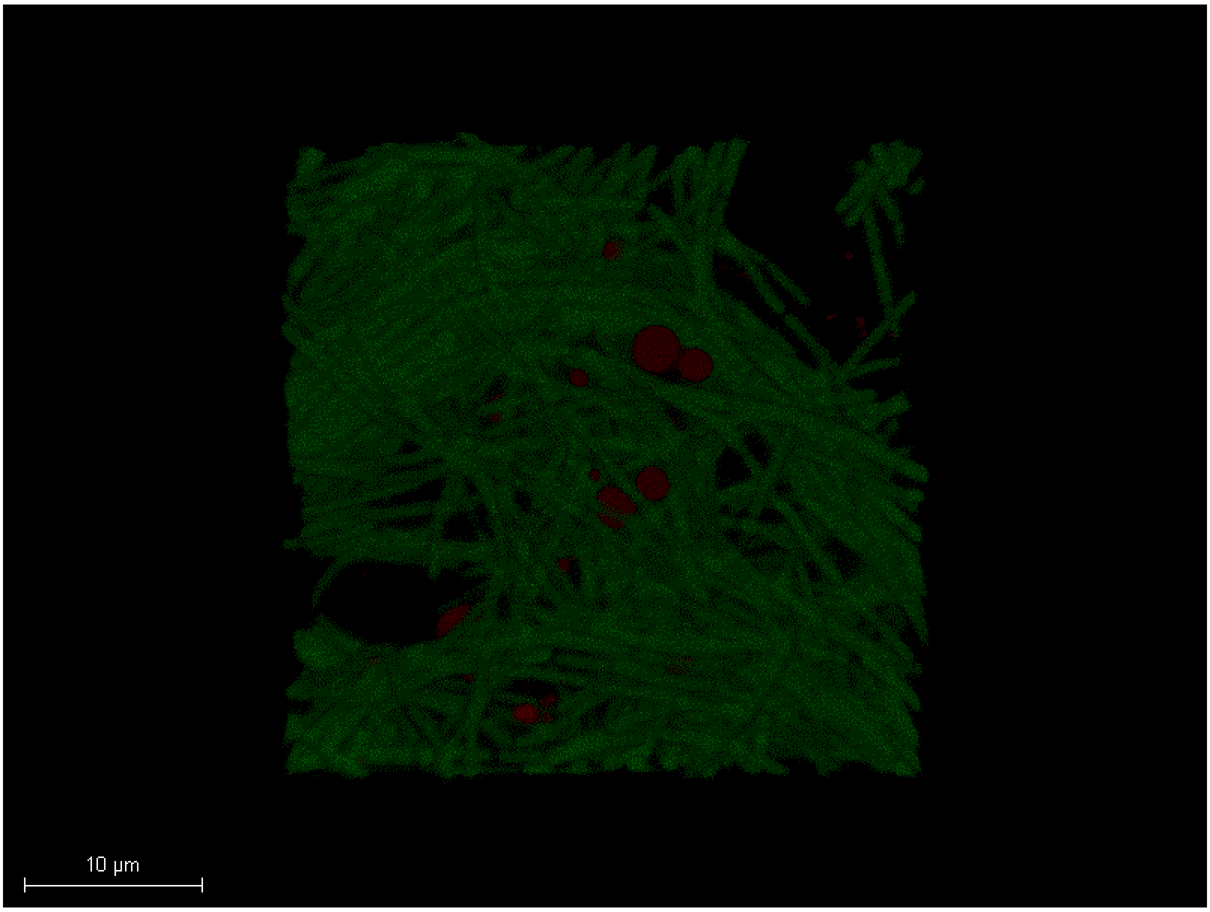

Confocal micrograph of *B. subtilis* expressing GFP (YC161) incubated with small MFG fraction for 24 h at 23ºC at 150 rpm. The MFG were stained with Nile red.
